# Some Observations on Simulated or Feigned Diseases

**Published:** 1824-02

**Authors:** A. Copland Hutchison

**Affiliations:** Surgeon to his Royal Highness the Duke of Clarence; senior Surgeon to the Westminster General Dispensary, and to the Royal Infirmary for the Diseases of Children; Surgeon to the Royal Naval Hospital at Deal during the last War, &c.


THE LONDON
Medical and Physical Journal.

2 OF VOL. LI.] FEBRUARY, 1824. [No. 300.

For many fortunate discoveries in medicine, and for the detection of numerous errors, the world is indebted to the rapid circulation of Monthly Journals; and there never existed any work, to which the Faculty, in Europe and America, were under deeper obligations, than to the Medical and Physical Journal of London, now forming a long, but an invaluable, series.—RUSH.

### ORIGINAL COMMUNICATIONS,
### SELECT OBSERVATIONS, &c.

ART. I.—*Some Observations on Simulated or Feigned Diseases.* By A. COPLAND HUTCHISON, Esq. Surgeon to his Royal Highness the Duke of Clarence; senior Surgeon to the Westminster General Dispensary, and to the Royal Infirmary for the Diseases of Children; Surgeon to the Royal Naval Hospital at Deal during the last War, &c.

IN a late valuable work on Medical Jurisprudence, published by Dr. PARIS, and a barrister of equal celebrity in his profession, J. S. M. FONBLANQUE, Esq., an interesting account is given of simulated or feigned diseases. It embraces the greater part of all that has been written on the subject, but adverts more especially to the works of Mahon, Foderé, and Dr. Hennen, of our own country.

The subject appears to me to possess much deeper interest than at first sight might be supposed, particularly in a national point of view; for it regards both the efficiency of men for the service of the state in times of war, and the protection of the real and innocent sufferer from suspicion. It is likewise important, in as much as an enlarged knowledge of this subject may enable the professional attendant to relieve the torturing anxiety of relatives, whose feelings are but too frequently worked upon by individuals of their own family, for the purpose of accomplishing some particular end; and, lastly, as it regards the practitioner,—for on the correctness of the medical man's prognosis will greatly depend his professional character.

It is for these reasons, chiefly, that I have deemed it a duty incumbent on me to offer such observations on this subject as the extensive experience my situation in the naval service has enabled me to make. I shall, therefore, without further comment, proceed to the subject in question, only requesting that such as may be sceptical, as to the great utility of this description of practical knowledge to the public and to the profession, may read the able and well-written exordium contained in Dr.

Paris's work above adverted to, (vol. i. p. 355,) where the most convincing arguments are adduced in proof of its importance, in various points of view.

*Ulcers.*—There is no species of imposture more practised by seamen, in order to obtain admission into naval hospitals, and eventually to evade the service, than that of making ulcers on the legs, or of extending and keeping open such as already exist.

The means pursued by these men are various. Such ulcers of this class as have come under my observation, have consisted in one or other of the following:—Cutting off a piece of skin with scissars, or with a knife; by the application of a blister, or quick lime, to the sound skin; or by the use of a mineral acid. When any part of the surface has been thus abraded, the sore is kept open and extended by the re-application of some of the above-named articles, or by the use of common copper coin: but, of all the substances resorted to on such occasions, that of acids is by far the most difficult to detect; for the impostor is often so alert, that, the surgeon's dressings being removed during a certain period, the acid acts upon the parts in dressings of his own, substituted in their stead, and, after the desired effect is produced, the ulcer is well washed, and the former dressings and bandages re-applied, with a care and cunning which renders a discovery very difficult. Other substances than the acids may sometimes be detected, by strictly observing the surface of the ulcer and the old dressings.

I recollect amputating the leg of a man at Deal Hospital, for a caries of the tibia, extending to the ankle-joint and upwards to the knee. This man asserted that he had never "played any tricks" with his leg, although I was persuaded that he had; and in this he persisted until the day after his leg was removed, when I showed him a piece of copper coin, which I had that morning discovered on dissecting the amputated limb, imbeded between the gastrocnemeus and soleus muscles, nearly three inches from the margin of the ulcer. The unfortunate man then distinctly stated, that he had thrust the piece of money into the ulcer, about nine months before, with the view of obtaining his discharge by invaliding; and this man lived to lament his imprudence.

As a remedy or preventive against such impostures, it has long been a practice in the navy and army to trust to a well-applied bandage, from the toes upwards to the knee, to seal the end of the roller with wax, and to impress it with the surgeon's own seal; but it was not long ere I discovered that this method was totally inefficient, by frequently observing a coagulum of blood upon the surface of the ulcer, and the surrounding skin in a state of inflammation, notwithstanding that the bandage and dressings remained apparently entire and untouched. This occurrence was rather difficult to account for, unless by the person striking the ulcered part against some hard substance: I afterwards, however, discovered, and it was confessed to have been done, by introducing a needle or pin through the bandage, and thus scratching the surface of the wound.

To guard, therefore, against this new device, I had a certain number of strong oaken boxes, made in the shape of a large square boot, to come up about four or five inches above the knee, the short thigh part of the boot forming with the leg an obtuse angle, so that the muscles of the diseased leg might be preserved in a relaxed or bent position. The upper end of this wooden boot was closed by a square piece of the same strong wood, with a circular hole cut in it to suit the circumference of the thigh, which was lined with list or leather, to prevent the cut edge of the wood from giving pain or uneasiness to the wearer. This boot, so made, is cut down the middle to the toe-part; hinges are then put on, and a lock, which cannot easily be picked, attached to the centre of the leg. Two narrow slits are then made through the sole of the boot, one on each side of the hollow of the foot, through which a leather strap is passed, for the purpose of being attached to a circular one round the ankle of the impostor, by means of two buckles, which, being inside, will completely preclude the possibility of the person's drawing his leg through.

When such characters came under my care in Deal Hospital, this was the plan pursued; and I never failed to heal all ulcers, after this measure was adopted.

In addition to what has been stated, it becomes necessary to mention that I had painted upon the lids or fronts of these boxes, in large letters, "*Punishment for Impostors;*" and in each ward, when they were not in use, we had two of them placed conspicuously, as a warning to all newly-admitted patients.

This, and other plans adopted, so completely succeeded in detecting impostures, which at this time were very common, that it soon spread among the crews of the different ships composing the grand fleet, then employed in the blockade of the Scheldt, as well as among the Downs squadron, under the distinct command of the port admiral; and, eventually, so notorious did Deal Hospital become for the detection of imposture, that latterly it was a very rare occurrence for such patients to be presented for admission; by which means, those fleets, then of such importance to the vital interests of Great Britain and her allies, were kept better manned, and therefore more efficient, than they could possibly have been, had the seamen found it easier to practise deceptions upon the surgeon.

*Diarrhœa.*—I have known diarrhœa and dysentery many times induced in hospital practice, where the individual found that he could not succeed in attaining the object of his desire, —viz. invaliding, by any other means; and, not unfrequently, I have witnessed such unfortunate men fall a sacrifice to their own imprudence, by inducing a disease which they would most gladly have been relieved from, at a period when perhaps it was too late. On this subject, the writer cannot readily forget the death-bed disclosures, and the pangs of conscience, he has witnessed on one or two occasions.

The bowel-affections, to which we now allude, can, I have reason to believe, be induced by various means, but that which was most resorted to by deluded seamen was a mixture of vinegar and burnt cork,—in what proportions I have never been able to ascertain: neither can I discover in what manner the burnt cork can assist in producing the disease in question; but certain it is that these were the ingredients most commonly had recourse to; and as certain is it that some of the finest young men in the navy have fallen victims to the use of such substances, for the accomplishment of their unhappy purposes. I have also known convicts in the Penitentiary at Millbank, previous to the late malady breaking out, both in their cells and in the infirmary, break down, with their fingers, in their urinary utensils, a good figured or formed motion, and intimately mix it with the urine, so as to induce the belief that it was in reality a diarrhœal evacuation; but a little attention to the character and appearances, with strict watching, will in most cases lead to a detection of the imposture. The object which convicts have in such practices is to be exempted from labour, and to be kept in the infirmary, where their comforts in every way are certainly much greater than when in their cells in the prison.

*Fever.*—This is another disease which I have known feigned. The particulars of the most remarkable case of it which came under my observation, I shall content myself with relating.

In 1801, I was appointed to the depôt for prisoners of war at Stapleton, near Bristol: in this new appointment it became my duty to go through the prison occasionally, (where there were between three and four thousand men,) for the purpose of selecting the sick for removal into the hospital, and where, as has already been stated, there is better fare and better accommodation than in the prison. Under such circumstances, indeed, the sick enjoyed the same comforts, to their fullest extent, as were granted by the government to British seamen in a naval hospital, which is such an ample supply of both food and wine as the medical officer may direct; and an hospital-dress, of both cloth and linen, is also invariably supplied to each patient, on his admission into the hospital. It is not to be questioned, therefore, that admission into the hospital became an object o[illegible] no small solicitude among those captive men, for they have no[illegible] unfrequently been known to gamble away, not only their clothes from off their backs, but even the last blanket in their hammocks, and the winner to barter them to persons who were constantly, at that time, given access to at the gate of the prison, or otherwise to carry on this traffic by throwing ropes over the walls, after the bargain was concluded at the gate, for the exchange of the articles.

In one of my daily visits in the prison, I was requested, with much earnestness and expression of concern, to visit a man in his hammock, who was stated to be labouring under high fever: his pulse was small, and so rapid that I could hardly count it; his tongue was covered with a brown coating, the eighth of an inch thick; and withal he was vomiting violently. Tobacco was strongly smelt in what he rejected from his stomach; and, on his recovering a little, I requested again to examine the tongue, when I removed a considerable portion of the soft and apparently foul substance, and brought it away with me on a piece of paper, for the purpose of examination: it proved to be common brown soap.

The man was conveyed to the hospital, narrowly watched for a few hours, at the end of which time his fever had entirely subsided; when a full acknowledgment was made by him, that it had been a common practice to obtain admission into the hospital by using tobacco and soap, as has been just described. But this discovery nearly cost me my life; for the next time I professionally visited the prison, this man made a thrust at me with a sword-stick, which, but for another prisoner who stood close to me, would certainly have passed through my body.

After this I never entered the prison but with four soldiers as a guard; when this man, on some of these occasions, assisted by a few of his friends, revenged themselves by picking vermin *(pediculi)* from their bodies, and blowing them through quills upon me as I past; so that I have frequently returned to my apartments covered with them.

*Contractions of the Hands, Elbows, and Knees; and of loss of Power, or Paralysis, of these parts.*—This imposture is, next to ulcers, the most frequent which we meet with among seamen and marines; and it must appear quite clear that a man, labouring under any of the ailments at the head of this section, will, so long as it is believed to exist, not only be excused from duty, but, if it continue, and the designs of the individual be not detected by the surgeon, he may eventually be invalided from the service, and be rewarded with that pension which is due, only, to real sufferings and services.

The first case of this kind that occurred to me was at the period when I was surgeon of the Druid frigate, commanded by that excellent officer, Sir Philip Broke, Bart. In the year 1806, the cook's mate of the ship had a contracted arm, and so immovable was the joint at the elbow, that the ulna and humerus had the appearance of being anchylosed; and, from long want of use, the muscles of that limb were so much reduced in size, that the arm had altogether an emaciated appearance. The man had never received any injury upon the arm, but accounted for it by a constant pain which he stated to have suffered all over that member, for several months previously to my having joined the ship; and during all this period he had been excused duty. I felt satisfied, after a few days, from a variety of concurring circumstances, that he was an impostor, and so reported him to the captain. But, notwithstanding that I continued so to report him for upwards of two months, that good man (for, in whatever ship Captain Broke sailed, he was the father of his people,) would not follow my advice, and make an example of him; which at that time, from the great number of such characters in the ship, was so necessary. The captain alleged, that I might possibly be mistaken, and that he should never forgive himself if he were to punish a man who afterwards might be found to be innocent of any crime. Upon this, I asked Captain B. whether, if I were to show the man's arm to him, and to the whole ship's company, rendered quite straight, without using force, he would then be convinced? His reply was in the affirmative.

After having acquainted Captain Broke with the plan I intended to pursue, he, in furtherance of this arrangement, ordered all hands on deck for punishment. When the man was stript for this purpose, I examined the contracted arm with great attention and minuteness, never making any attempt to stretch it out, until I saw the influence of the will taken off from the muscles of his arm, by his earnestness and great anxiety to answer some question put to him by the captain, who all the while I was so employed, (about ten minutes or a quarter of an hour,) kept engaging his attention in conversation, in order to divert his volition from the contracted arm: then, to the utter confusion of the cook's mate, and with indignant expressions of "Shame!" "Punish him!" echoing from nearly the whole crew, the arm was, without the slightest effort, made perfectly straight; and such was the effect it had upon the man himself, that, unconsciously, he raised the hand of the now-restored arm to his forehead, and intreated forgiveness. He was punished, and went immediately to his duty, without ever complaining again during the two years I remained in the ship; and such was the effect of this example upon the minds of the men, that very few skulkers ever troubled us again.

An excellent seaman, six feet high, was admitted under my care at Deal Hospital, labouring under a paralysis of the right arm. Circumstances connected with this man's case led me to suspect him to be an impostor; and, after nearly two months' residence in the hospital, immediately before a survey for invaliding was to take place, I caused fifty drops of tinctura opii to be administered to him in his tea or supper, unknown to him. At eleven o'clock that night, I visited him, accompanied by four hospital mates, and one nurse from each ward. Most of the patients in his apartment, which contained fourteen, were asleep. I approached this man's bed, stood up against the wall on his right side, and tickled his right ear with a feather; when, to the astonishment and mirth of the assembled party, the paralysed hand was instantly raised to his ear, which he rubbed with no small degree of force, and then turned round upon his left side, dragging the bed-clothes over him with his heretofore useless arm. He was not aroused, there being only the usual light in the ward, a common rush-candle; and, when he was again supposed to be asleep, the same operation of the feather was repeated, but with greater irritation; the paralysed arm was again raised as before, and, from the loud laughing that now prevailed, he was instantly aroused, and, finding himself detected, he sprung out of his bed, and caught me with both arms round the neck, and said in a whisper, "I hope I shall meet with you, sir, some day in a dark corner." This man was sent to his ship, and I afterwards learnt that he zealously performed his duty.

Another man was brought to the hospital, for the purpose of being invalided, on the day when the surveying officers were assembled upon this service. The patient was stated to have lost the use of the extensor muscles of the right hand, so that this member was always found hanging down, apparently powerless. It was winter; and my colleague, one of the surveying officers on the occasion, suggested that the hand, in its relaxed and useless state, might be placed over the edge of the table round which we were all seated, and some assistants, who stood by, were directed to keep the arm and shoulder firmly fixed, so that the patient might not be able to withdraw his hand from that situation: my colleague then turned round to the fire, took from the grate a red-hot poker, and placed the heated point near the extremity of the insertion of the flexor tendons, on the inner surface of the fingers. The hand was then gradually seen rising, as the hot poker approached it, until the extensor muscles had raised it to its utmost. The man was returned to his ship *cured*, and performed his duty as formerly.

This part of the subject might be illustrated by numerous other instances, but those that have been here advanced, I am of opinion, are sufficient to put the medical officer upon his guard, when placed under similar circumstances.

*Inflammation of the Eyes.*—Various are the instances of the loss of vision which I have witnessed from mal-practices on this organ, and numerous are the individuals who have received pensions for the injuries they have themselves produced.

In the year 1801, whilst serving as surgeons' mate on-board the Alkmaar hospital-ship in the Baltic Sea, a patient laboured under the most active ophthalmia I had ever then witnessed. It was soon discovered, however, that the disease was much less acute during the intervals of the visits of the physician to the fleet, Dr. Baird, and the surgeon of the ship, Mr. Jamieson. This led to some inquiry and investigation, when, on a strict examination of the bed and person of the patient, was found concealed under the pillow a paper containing powdered alum, which the unhappy man had more than once been seen to introduce into his eyes. One eye was totally lost by this circumstance; and three years afterwards the writer met the identical person in Darkhouse-lane in London, and was informed by him that, "Although you would not favour my views, you see I have at length got rid of the service; and have, besides, obtained a pension for the loss of my eye."

Several such cases occurred to me at Deal Hospital. Alum, lime, and tobacco-juice, are the substances most generally used to produce this disease; and we found no better remedy than to confine the patient's hands by the application of a straight waistcoat, a strict watch to be kept over him, and a perseverance in the common treatment to subdue inflammatory action in this organ.

*Incontinence of Urine.*—Numberless are the instances of this deception on-board ships of war, and numberless are the brave men who have been lost to the naval service by invaliding from this cause.

After having attentively examined the urethra and bladder, by means of a sound or staff, the method I have found most effectual in detecting this imposture has been to administer a large dose of opium, and, when the patient is sound asleep, to place gently under him a clean dry folded sheet, and which is to be frequently examined during the night, by the nurse of the ward, or by an hospital-assistant, with the view of ascertaining whether the sheet remain dry whilst the patient is asleep, and under the influence of the narcotic. If the sheet remain dry for four or six hours, and be only found wetted after he has awoke, the natural conclusion must be that the individual is an impostor; and I have no hesitation in stating this to be my opinion, without referring it to the influence of the medicine.

*Stricture.*—It is very painful, although quite necessary in this place, to state, for the information of the medical officer in the king's service, that, in hospital practice, we are frequently called upon to examine officers, who are presented for admission into naval hospitals, in consequence of some disagreement with their captain or messmates; and stricture is more frequently the disease feigned by them on such occasions than any other: but, by this remark, it is by no means meant to be inferred that the majority of cases of this kind are feigned: on the contrary, there are certainly numerous instances of this disease among officers, that are, unhappily for the individuals, but too well verified.

When an officer has been presented at Deal Hospital under these circumstances, it was my uniform practice to place the patient's back against the wall, so as not to admit of retreat. A bougie was then passed into the urethra, when, in many cases, the passage of the instrument beyond the perinæum has been very difficult to accomplish. To ascertain whether this stop were really a stricture, or merely a voluntary constriction of this part of the canal by the neighbouring muscles, the following has been the plan adopted to decide the question. On the arrival of the bougie at this place in the urethra, gentle pressure is made with the instrument, by moving the bougie somewhat quickly backwards and forwards against the stricture, and yet without using any force. The patient, during this period, is to be engaged in conversation with the surgeon, as to the ships, climates, and battles, he has served in,—the captains' names of his respective ships,—his part of the country, relatives, prospects of advancement in his profession, &c.; and, when there is reason to suppose the mind at any time to be taken off from the operation, a gentle movement of the instrument forwards will, if there be not real stricture, readily pass it on into the bladder.

In this way a case of simulated stricture may be detected, nine times out of ten; and immediately that such officer has been pronounced as not labouring under this disease, the true state of the case has occasionally been mentioned,—namely, that a disagreement existed in his ship; and that, if he had not made use of this plea, a court-martial may have been the issue, and possibly the loss of his commission.

The naval-hospital surgeon, under such circumstances, has a very difficult and serious duty to perform; and I hardly know of any situation, at least in some instances which came under my own observation, more distressing to the feelings than the one now adverted to: yet there is a paramount duty to which we are pledged, and from which we cannot, in conscience, as servants to the public, deviate. It is the straight-forward course, in all such cases, that ought to be pursued.

Spasmodic stricture, as it is commonly called in private practice, may generally be distinguished from strictures of a permanent nature, by the means above described.

*Hernia.*—In examining new recruits, either for the army or navy, it is essential that they be free from hernia, both on account of their efficiency, and to preclude the possibility of seamen afterwards making a pretext of being ruptured in the service, and thereby obtaining *smart-tickets*, which will entitle them to pensions for life: and, as many ruptures are easily reduced, it has been, to my knowledge, not an unusual practice for such men to appear, on examination, to be perfectly free from this affection.

Recruits should therefore be examined naked, and should be made to jump, and to cough as violently as they can, by which means a hernia, if it do exist at all, will in most cases become sufficiently apparent to authorise the surgeon to reject them.

I have one or two remarks more to offer, before I take leave of this subject. No class of men are more subject to hernia than seamen, from the great bodily exertions they are frequently called upon to make, either in furling sails in a gale or in heaving-up the anchor, besides various other severe duties; and yet I have never once found it necessary to perform the operation for strangulated hernia upon a seaman. I have never seen a case of it, indeed, among this class of men, excepting in one solitary instance, and this was not known until dissection showed it after death. It happened to be a singular case of strangulation of the colon through the diaphragm. I have learnt, also, from other naval-hospital surgeons, that it is very seldom they have had occasion to perform this operation. The great and repeated distention of the abdominal apertures in seamen who are affected with hernia, by the nature of their occupations, as above noticed, may possibly in some degree account for it; or it may be accounted for by the constant residence among them of a medical officer, (to whom seamen are always most ready to apply for relief, when in any way indisposed,) and who, being early applied to, may, in many instances, have succeeded in reducing the hernia, which, but for such timely aid, might have become strangulated.

Hernia occasionally concealed by seamen, for the purpose of procuring a pension, and of being afterwards invalided to enjoy such pension in some other situation, as in the merchant service, where they are paid according to the work they are capable of performing, without reference to any disease with which they may be affected; the existence of hernia among such men being well known to be an insuperable bar to their admission into the British navy. I have a curious physiological fact, illustrative of this, to relate to the professional reader, and which came under my own personal observation.

In July, 1806 or 1807, during a cruise off the Island of Madeira, in H. M. S. Druid, a merchant ship from the coast, bound to Glasgow, was boarded, from which an English seaman and a negro boy were impressed, and, according to the custom of the service, were, previously to their being finally detained, ordered to the cockpit for the purpose of examination, by the surgeon, as to their fitness for the king's service. The man, on stripping himself, said that he was ruptured in both groins; that he had been "overhauled" (examined) a dozen times by the surgeons of different ships of war; and that he had as frequently been discharged again as unserviceable, from this cause.

There certainly was a swelling in each groin, very much resembling hernia; but the weather at this time being extremely hot, and the scrotum therefore very pendent and flaccid, my attention was particularly called to it; and, on examination, I found the scrotum to be an empty bag, and the testes (of their natural size) lodged in the groin. As soon as this discovery was made, the poor man, from being at length and so unexpectedly detected, became quite unnerved, and so agitated, that, upon re-examining the parts, the testes were found to have descended into their proper places in the scrotum. After commending the man for his ingenuity, and, in place of physic, administering to him a glass of grog, his spirits were rapidly restored, and seeing no longer any chance of eluding the king's service, he displayed before us several very remarkable feats of power he possessed over these organs. He pulled both testes from the bottom of the scrotum up to the external abdominal rings, with considerable force, and again dropt them into their proper places, with incredible facility. He then pulled up one testis, and, after some pause, the other followed, as the word of command was given; he then let them both drop into the scrotum simultaneously. He also pulled one gradually up, whilst the other was as gently descending; and he repeated this latter experiment as rapidly as the eye could well follow the elevation and descent of the organs, so that my assistant and myself were not only surprised, but so exceedingly amused, that we could hardly believe the evidence of our senses. This man afterwards proved to be a willing, hard-working man.

Except in the above remarkable case, I know of no instance on record of the cremaster muscles being muscles of will; and, as it was so singular a case, I showed him to several of the medical officers of the squadron we happened to meet with, during the remainder of my service in that ship.

I must here break off rather abruptly, the above being all I have at present leisure to communicate: at a future period I may, perhaps, follow up this subject in another Number of your useful Journal. It is hoped, however, that enough has been here stated to put the medical officers in his Majesty's service sufficiently on their guard to enable them to detect such abuses as have been described; at the same time, that all due consideration and feeling should most scrupulously be exercised by my professional brethren, on so very delicate and important a duty as that which has been the subject of this paper.

As it may be apprehended that I may have over-stated the frequency, and over-rated the detriment, which such feigned complaints have produced in the king's service, I beg to subjoin the following letter, *verbatim et literatim*, from one of the most impudent impostors that ever came under my notice.

"To A. COPLAND HUTCHISON, Esq. Surgeon, Royal Naval Hospital, Deal.

"*June* 21, 1810.

"Sir,—I beg your pardon for taking the liberty of writting to you. Sir, all the information I can give you against that man, is* that I know him to bring stuff to James Chapman, but I do not know the name of it. He brought dragon's blood and oil of vitriol to James M'Donald, that was in the 13th ward. He brought vinegar and cork-wood, which was burned and mixed together, and taken by Handover in the 13th ward. There was some stuff brought to Gego, that he used to his leg, and Pattison. There was many more got things of this kind, but I am not sure of them. But all I have said I am sure of. He has taken out many suits of sailors cloths, and sold them for to by these things and licker. Sir, it would be better for I had cast myself into the sea than told you of this, if you let it be known, for every one that does not lake the sarves would think little of my life.

"The only thing that made me speak of it was, that I thought you used every one under your care well, and I am sure that man did currupt many in hospital, and hurt much of your handy work. Sir, I am on board the gard ship at the Noar, and expects to be sent to you. Richard Hobdey is the man. All from your homble servant,

(Signed) "JAMES MITCHELL."

In palliation of the moral turpitude which truth compels me to impute to our brave defenders, be it recollected that the late revolutionary war was one of unexampled length, in which human endurance was put to the most severe trial. From the year 1793 to 1815, the efforts of the navy were unremitting, except a short breathing after the peace of Amiens; and, of all lives, one constantly engaged either in long dreary voyages, cruises,

* This man (Mitchell) had written before, to say that a labourer belonging to the establishment was much employed by the patients in the hospital, in the manner above explained; and the above communication was in answer to a letter I had written to him, to name the person. Inquiry proved the correctness of Mitchell's statement, and Hobdey was forthwith dismissed from his office.

and blockades, or in combating the elements and the enemy, tends most to wear out patience, not only through the hardships, dangers, and ennui, inseparable from them, but by the long absence from friends, kindred, and sweethearts; so that some of the best feelings of our nature are arrayed against the sense of public duty, and plead loudly in extenuation of the offence which is the subject of this article.

*Spring Gardens; December* 1823.

