# Erratum

**Published:** 1824-02

**Authors:** 


Erratum.—In page 96, six lines from the bottom, read " Hernia is," &c.

